# Real-Time Blood Flow Assessment Using ICG Fluorescence Imaging During Hepatobiliary and Pancreatic Surgery with Consideration of Vascular Reconstruction

**DOI:** 10.3390/cancers17050868

**Published:** 2025-03-03

**Authors:** Hiroyuki Fujimoto, Masahiko Kinoshita, Changgi Ahn, Takuto Yasuda, Kosuke Hatta, Mizuki Yoshida, Koichi Nakanishi, Takahito Kawaguchi, Naoki Tani, Takuma Okada, Genki Watanabe, Ryota Tanaka, Shigeaki Kurihara, Kohei Nishio, Hiroji Shinkawa, Kenjiro Kimura, Takeaki Ishizawa

**Affiliations:** 1 Department of Surgery, Osaka Metropolitan University Hospital, 1-4-3 Asahimachi, Abeno-ku, Osaka 545-8585, Japan; hiroyuki530930@icloud.com; 2 Department of Hepatobiliary-Pancreatic Surgery, Graduate School of Medicine, Osaka Metropolitan University, 1-4-3 Asahimachi, Abeno-ku, Osaka 545-8585, Japan; o23709k@omu.ac.jp (C.A.); u21572m@omu.ac.jp (T.Y.); s23780n@omu.ac.jp (K.H.); j23328c@omu.ac.jp (M.Y.); d23100k@omu.ac.jp (K.N.); j22652b@omu.ac.jp (T.K.); y21907s@omu.ac.jp (N.T.); i21455a@omu.ac.jp (T.O.); w24247k@omu.ac.jp (G.W.); x23994z@omu.ac.jp (R.T.); j23142w@omu.ac.jp (S.K.); u21474n@omu.ac.jp (K.N.); d21129t@omu.ac.jp (H.S.); v21873r@omu.ac.jp (K.K.); take1438@gmail.com (T.I.)

**Keywords:** indocyanine green fluorescence imaging, vascular reconstruction, real-time blood flow assessment, hepatobiliary and pancreatic surgery

## Abstract

Background/Objectives: Indocyanine green (ICG) fluorescence imaging is widely utilized for visualizing hepatic tumors, hepatic segmentation, and biliary anatomy, improving the safety and curability of cancer surgery. However, its application for perfusion assessment in hepatobiliary and pancreatic (HBP) surgery has been less explored. Methods: This study evaluated outcomes of patients undergoing HBP surgery with vascular reconstruction from April 2022 to August 2024. During surgery, ICG (1.25–5 mg/body) was administered intravenously to assess the need and quality of vascular reconstruction via fluorescence imaging. Results: Among 30 patients undergoing hepatectomies and/or pancreatectomies, ICG fluorescence imaging was used in 16 cases (53%) to evaluate organ and vascular perfusion. In two hepatectomy cases with consideration of reconstruction of the middle hepatic veins, sufficient fluorescence intensities in drainage areas led to the avoidance of middle hepatic vein reconstruction. In 14 cases requiring vascular reconstruction, fluorescence imaging visualized smooth blood flow through anastomotic sites in 11 cases, while insufficient signals were observed in 3 cases. Despite this, re-do anastomoses were not indicated because the fluorescence signals in the targeted organs were adequate. Postoperative contrast-enhanced computed tomography confirmed satisfactory blood perfusion in all cases. Conclusions: Real-time blood flow assessment using ICG fluorescence imaging provides valuable information for intraoperative decision-making in HBP surgeries that require vascular reconstruction of major vessels, such as hepatic arteries, veins, and the portal system.

## Simple Summary

Indocyanine green (ICG) fluorescence imaging is widely employed in hepatobiliary and pancreatic (HBP) surgery; however, its effectiveness for intraoperative blood flow assessment in HBP procedures involving major vascular resection has been sparsely reported. This study evaluated 16 cases where ICG fluorescence imaging was used for real-time evaluation during HBP surgery with vascular reconstruction. In two cases, reconstruction was avoided based on ICG fluorescence evaluation. Among three cases with insufficient fluorescence signals in reconstructed vessels, one underwent re-do anastomosis after reconstruction, guided by fluorescence imaging. This approach may contribute to intraoperative decision-making for vascular reconstruction and re-do anastomosis based on real-time blood flow assessment.

## 1. Introduction

Extended surgery with vascular reconstruction is commonly performed for advanced cancers across various surgical fields. In hepatobiliary and pancreatic (HBP) surgery, pancreaticoduodenectomy (PD) involving portal vein (PV) or superior mesenteric vein (SMV) resection carries higher risks of postoperative complications and mortality, along with lower survival rates, compared to PD without vascular resection, as shown by meta-analyses [1,2,3]. Similarly, in liver resection, particularly during liver transplantation and surgery for perihilar cholangiocarcinoma, vascular reconstruction or occlusion at reconstructed sites is associated with poorer postoperative outcomes compared to procedures without vascular reconstruction [4,5,6]. Despite its necessity for achieving curative resection, vascular reconstruction poses a significant risk of severe complications, such as impaired blood flow or thrombus formation in reconstructed vessels [7]. Accurate intraoperative assessment of reduced blood flow caused by stenosis or bending at reconstructed sites may help improve postoperative outcomes in patients undergoing HBP surgery with vascular reconstruction.

The effectiveness of real-time blood flow assessment using indocyanine green (ICG) fluorescence imaging has recently been demonstrated in various fields, including plastic surgery, gastrointestinal anastomosis, breast surgery, and coronary artery reconstruction [8,9,10,11,12]. Additionally, its utility in assessing anastomotic perfusion in colorectal surgery has been highlighted [13]. However, while ICG fluorescence imaging is widely applied in HBP surgery, it is primarily used for fluorescence cholangiography to visualize bile ducts, tumor localization, and hepatic segmentation [14,15,16,17,18,19,20,21,22,23]. Reports on its application for blood flow assessment in HBP surgery are limited to specific cases, such as vascular anastomosis in liver transplantation [24] and blood flow evaluation during left gastric artery reconstruction in distal pancreatectomy (DP) with celiac axis resection [25]. Assessing blood flow in reconstructed vessels or target organs during HBP surgery can play an important role in the prevention of critical postoperative complications. Therefore, the efficacy of ICG fluorescence imaging in this context warrants further investigation.

This retrospective study aims to evaluate the utility of ICG fluorescence imaging for real-time blood flow assessment in HBP surgeries requiring vascular reconstruction.

## 2. Materials and Methods

### 2.1. Study Cohort

This study involved 30 patients who underwent HBP surgery with major vascular resection and potential vascular reconstruction at Osaka Metropolitan University Hospital, Osaka, Japan, between April 2022 and August 2024. To evaluate the utility of ICG fluorescence imaging for blood flow assessment, the association between the presence of fluorescence signal in reconstructed vessel or surrounding organs and perioperative outcomes including postoperative vascular complications were analyzed in these patients who underwent ICG fluorescence imaging during surgery. All surgeries requiring major vascular resection were conducted via open surgery. Decisions regarding vascular resection and/or reconstruction were based on imaging studies and reviewed during a preoperative conference attended by six board-certified HBP surgeons in Japan.

### 2.2. ICG Fluorescence Imaging and Surgical Procedures

The use of ICG during surgery was determined at the discretion of the operating surgeon.

In our department, 1.25–5 mg/body of ICG was administered intravenously to evaluate blood flow in reconstructed vessels or targeted organs. Real-time blood flow assessment was performed using ICG fluorescence imaging devices, either the Rubina Lens^®^ (Karl Storz, Kennesaw, GA, USA) or LIGHTVISION^®^ (Shimadzu Corporation, Kyoto-Shi, Japan). The sufficiency of fluorescence signals in the targeted organ or reconstructed vessel was evaluated intraoperatively by at least two board-certified HBP surgeons in Japan. Based on these evaluations, decisions were made regarding the necessity of vascular reconstruction or re-do anastomosis after reconstruction, with consideration of the fluorescence signals in the vessel or targeted organs.

Vascular reconstruction techniques were classified according to the standardized terminology proposed by the International Study Group of Pancreatic Surgery for vascular resection [26]: type 1, partial venous excision with direct suture closure; type 2, partial venous excision using a patch; type 3, segmental resection with primary venovenous anastomosis; and type 4, segmental resection with an interposed venous conduit requiring at least two anastomoses.

### 2.3. Ethical Considerations

This retrospective study adhered to the ethical principles outlined in the Declaration of Helsinki. Ethical approval was granted by the Ethics Committee of Osaka Metropolitan University (Approval No. 2022-142). Written informed consent was obtained from all participants.

## 3. Results

Out of the 30 patients who underwent HBP surgery, 16 patients (53%) were evaluated for organ and vascular perfusion using ICG fluorescence imaging. Table 1 presents the clinical characteristics, ICG fluorescence imaging findings, and postoperative outcomes of these 16 patients. In these 16 patients, blood flow was assessed in 21 vessels and targeted organs were evaluated. The targeted vessels included the SMV (*n* = 10), right hepatic artery (RHA; *n* = 2), proper hepatic artery (PHA; *n* = 1), common hepatic artery (CHA; *n* = 1), middle hepatic vein (MHV; *n* = 1), anterior fissure vein (AFV; *n* = 1), right hepatic vein (RHV; *n* = 2), celiac artery (CA; *n* = 1), and splenic vein (SpV; *n* = 2).

**Figure 1:**
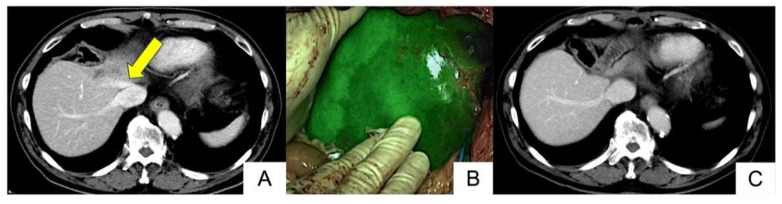
A case of reconstruction avoidance, using LIGHTVISION® (Patient no. 2). The metastatic liver tumor is located near the MHV ((**A**), arrow). Mild hepatic congestion was observed during ICG fluorescence imaging (**B**), but no hepatic congestion was seen in the contrast-enhanced CT after surgery (**C**). Supplementary Video S1 demonstrated the operative movie in patient no. 2.

In two hepatectomy cases where reconstruction of the MHV and AFV was considered, vascular reconstruction was avoided based on the ICG fluorescence imaging findings. In one case, an extended left hemi-hepatectomy with MHV resection was performed for a metastatic liver tumor (Patient no. 2, Figure 1A). ICG was administered after clamping the MHV, and mild hepatic congestion was observed in the remnant liver (Figure 1B). As a result, it was decided not to proceed with vascular reconstruction. A postoperative contrast-enhanced computed tomography (CT) scan confirmed the absence of hepatic congestion (Figure 1C). Supplementary Video S1 demonstrated the operative movie in patient no. 2.

In a case of partial hepatectomy with AFV resection (Patient no. 6), ICG was administered after clamping the AFV during surgery, and only mild hepatic congestion was observed in the liver perfusion area. As a result, AFV reconstruction was avoided. Postoperatively, no hepatic congestion was observed.

In contrast, vascular reconstruction was necessary in 14 patients (11 pancreatectomies, 2 hepatectomies, and 1 bile duct resection) involving 19 vessels. Of these, 17 vessels underwent type 3 reconstruction, and 2 vessels underwent type 4 reconstruction. In three reconstructed vessels (RHA, Patient no. 4; RHV, Patient no. 5; SpV, Patient no. 12), insufficient fluorescence signals were observed, representing 15% of all reconstructions.

In a case involving RHA reconstruction (Patient no. 4), a re-do anastomosis after reconstruction was performed due to an insufficient fluorescence signal. Intraoperative findings showed occlusion of the reconstruction site due to vascular intimal injury. After the re-do anastomosis, the fluorescence signal improved, and no hepatic ischemia was observed with ICG fluorescence imaging. Postoperatively, the patient developed thrombosis of the reconstructed RHA, classified as Clavien–Dindo grade I [27]. Although partial hepatic ischemia was observed, blood flow and hepatic perfusion improved without the need for antithrombotic therapy.

**Figure 2:**
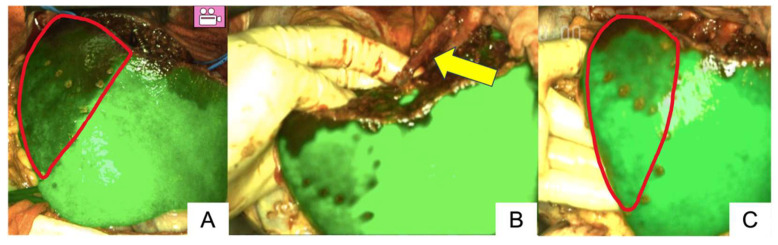
A case of insufficient fluorescence imaging, using LIGHTVISION® (Patient no. 5). Hepatic congestion was observed before the reconstruction ((**A**), circled area). The fluorescence signal at the reconstructed vessel site was insufficient ((**B**), arrow). Hepatic congestion improved after reconstruction ((**C**), circled area). Supplementary Video S2 demonstrated the operative movie in patient no. 5.

In a case involving RHV reconstruction (Patient no. 5), partial hepatectomy with RHV resection was performed for a metastatic liver tumor. ICG was administered after clamping the RHV, and hepatic congestion was observed before reconstruction (Figure 2A). ICG fluorescence imaging showed insufficient fluorescence at the site of type 4 vascular reconstruction using the umbilical vein (Figure 2B). However, hepatic congestion improved over time (Figure 2C), and no re-do anastomosis was performed. Postoperative contrast-enhanced CT showed no blood flow through the RHV, but hepatic blood flow was adequately drained via the MHV, with no hepatic congestion observed. Supplementary Video S2 demonstrated the operative movie in patient no. 5.

**Figure 3:**
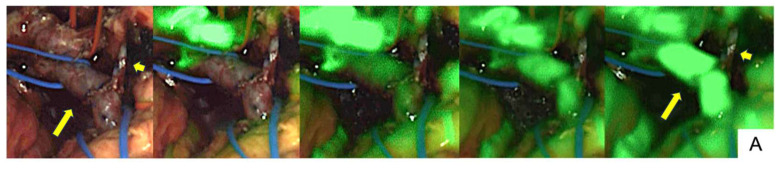

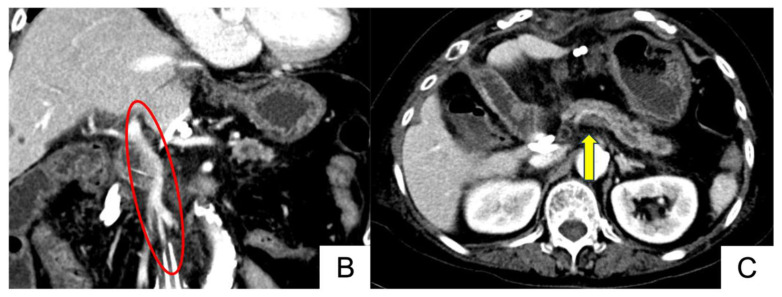
A case of sufficient and insufficient fluorescence imaging, using LIGHTVISION® (Patient no. 12). The time-series changes in ICG fluorescence signals were observed before ICG administration and at 10, 15, 20, and 30 s after administration (**A**). The ICG fluorescence signal in the reconstructed vessels was sufficient in the SMV ((**A**), arrow) but insufficient in the SpV ((**A**), short arrow). Blood flow in the SMV was clearly observed on contrast-enhanced CT ((**B**), circled area), whereas no blood flow was observed in the SpV ((**C**), arrow).

In a case involving SMV and SpV reconstruction (Patient no. 12), ICG fluorescence imaging showed sufficient fluorescence signals at the SMV reconstruction site, but insufficient fluorescence signals at the SpV suggested reduced blood flow (Figure 3A). Since blood flow through the SMV trunk was sufficient, re-do anastomosis of the SpV was not performed. Postoperative contrast-enhanced CT showed no thrombosis in the SMV (Figure 3B), but thrombotic occlusion was present in the SpV (Figure 3C).

**Figure 4:**
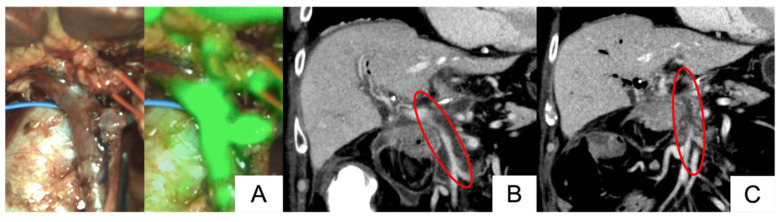
A case of sufficient fluorescence imaging with postoperative thrombosis, using LIGHTVISION® (Patient no. 15)**.** The ICG fluorescence signal in the reconstructed vessel was sufficient (**A**). Blood flow was observed in the reconstructed vessel on contrast-enhanced CT on postoperative day 3 ((**B**), circled area), but no blood flow was observed on postoperative day 19 ((**C**), circled area).

Among the 16 vessels with sufficient fluorescence signals in the reconstructed vessel, postoperative vascular thrombosis was observed in one case involving two vessels. A PD with SMV and SpV resection was performed for pancreatic cancer (Patient no. 15). ICG fluorescence imaging showed sufficient fluorescence signals at the reconstructed sites (Figure 4A). A contrast-enhanced CT scan on postoperative day 3 showed stenosis of the SMV but no thrombosis or occlusion (Figure 4B). However, a contrast-enhanced CT scan on postoperative day 19 revealed thrombotic occlusion at both the SMV and SpV reconstruction sites (Figure 4C). Despite this thrombotic occlusion, no hepatic ischemia was observed, and anticoagulation therapy with heparin was started.

In summary, vascular reconstruction was avoided in 2 of 16 patients (2 of 21 vessels) based on ICG fluorescence imaging findings. Among the three patients with insufficient fluorescence signals in reconstructed vessels, only one patient required a re-do anastomosis after reconstruction, while the other two patients did not, as sufficient blood flow was confirmed in the targeted organs based on ICG fluorescence imaging. Of the 11 patients (16 vessels) with sufficient fluorescence signals in the reconstructed vessels, postoperative thrombotic occlusion occurred in 1 patient (2 vessels), corresponding to an incidence rate of 12.5%.

## 4. Discussion

ICG fluorescence imaging relies on the fact that protein-bound ICG emits fluorescence signals when exposed to near-infrared light [28,29,30]. After intravenous injection, ICG is absorbed by hepatocytes and nearly 100% is excreted in bile. This property is utilized in various ways during fluorescence-guided surgery [31,32,33,34]. In HBP surgery, reports on ICG fluorescence imaging for evaluating vascular reconstruction sites and organ blood flow have mostly focused on blood flow assessment during PD with CHA resection and the evaluation of reconstructed vessels in liver transplantation. However, these are mostly case reports [35], and the relationship between fluorescence imaging findings and postoperative vessel patency remains unclear [36]. The latter application is limited to specific surgical techniques [37]. Given this background, real-time blood flow assessment during HBP surgery largely depends on intraoperative ultrasound sonography. The results presented here describe our experience using ICG fluorescence imaging to determine the need for vascular reconstruction and assess blood flow in reconstructed vessels and targeted organs. Few studies have reported on the use of real-time blood flow assessment with ICG fluorescence imaging in HBP surgery, making this study clinically significant.

Despite the indication determined by a detailed conference with several experts regarding the need for vascular reconstruction, 2 of 16 patients (2 of 21 vessels) avoided reconstruction based on ICG fluorescence imaging findings, without any postoperative complications. These cases involved hepatectomy with major hepatic vein resection. During hepatectomy, venous obstruction reduces PV inflow in the affected region, a phenomenon known as the veno-occlusive region [38]. Accurate intraoperative or preoperative assessment of the veno-occlusive region is crucial for determining the appropriate liver volume to retain postoperatively, contributing to safer hepatic resection [39]. Conversely, unnecessary hepatic vein reconstruction should be avoided due to the risk of pulmonary artery embolization if a thrombus forms in the reconstructed vessel [40]. These results suggest that ICG fluorescence imaging can play a critical role in intraoperative decision-making regarding the need for hepatic vein reconstruction.

Postoperative thrombotic occlusion was observed in 1 of 11 patients (2 of 16 vessels) despite sufficient fluorescence signals in the reconstructed vessels. This patient (Patient no. 15) underwent PD with SMV and SpV resection and reconstruction. PD is a common procedure for malignant tumors such as pancreatic and bile duct cancers, accounting for 63% (19 of 30 cases) in this study cohort. Due to the anatomical characteristics of these tumors, combined resection of the SMV is often necessary for curative resection, with reported rates of 19%–25% [41,42]. Postoperative thrombosis in reconstructed vessels is a major complication in PD with SMV resection [2,43]. In this study, the incidence of postoperative SMV thrombosis in cases using ICG fluorescence imaging was 11% (one of nine cases), which is similar to previously reported rates [41,42,43]. Many factors contribute to postoperative thrombus formation, including intraoperative blood loss and infectious complications [44,45]. In PD, delayed thrombosis may occur with inflammation due to postoperative pancreatic fistula [46]. While it may be difficult to prevent thrombus formation solely through intraoperative blood flow assessment, careful monitoring with ultrasound or early postoperative contrast-enhanced CT is necessary. These findings suggest that ICG fluorescence imaging has limited effectiveness in reducing the risk of postoperative thrombosis. However, in the three patients with insufficient fluorescence signals in the reconstructed vessel, one required re-do anastomosis after reconstruction. The postoperative course of this patient was largely uneventful. While ICG fluorescence imaging may not fully prevent thrombosis, it is a useful technique for decision-making regarding re-do anastomosis after reconstruction, potentially reducing the risk of postoperative complications.

The dosage of ICG may have impacted the fluorescence imaging and potentially affected blood flow assessment. Since there is no universally established standard dosage, the ICG dosage varied depending on the intended purpose. Previous reports on liver transplantation have used doses ranging from 2.5 to 25 mg per body weight or 2.5 μg/mL per liver graft volume [47]. For blood flow assessment during PD with CHA resection, a dose of 2.5 mg/body was used [25,35]. For liver segmentation, the typical dose is 2.5 mg/body, with reported ranges from 0.025 mg to 25 mg/body [48]. In this study, 1.25–5 mg of ICG effectively visualized blood flow in the reconstructed vessels or target organs, which is considered an appropriate dosage. However, the sensitivity of ICG fluorescence imaging can also be affected by the thickness of surrounding tissue, so the ICG dose may need to be adjusted in cases of inflammatory thickening [49].

As mentioned earlier, the results suggest that ICG fluorescence imaging plays an important role in intraoperative decision-making during HBP surgery that involves major vascular resection with consideration for vascular reconstruction. Although its effectiveness in preventing thrombosis in reconstructed vessels cannot be confirmed at this stage, it has the potential to improve postoperative outcomes. However, this study has several limitations. First, it was a single-center, retrospective study with a small sample size. It is notable that the results suggest the usefulness of this technique despite the limited number of cases, but further validation in larger studies is needed. Second, technical challenges persist in the application of ICG fluorescence imaging. A standard for quantitatively evaluating fluorescence intensity has yet to be established, and subjective factors inevitably influence intraoperative assessments [24,50]. In addition, research is being conducted to evaluate tissue perfusion by analyzing ICG fluorescence intensity using artificial intelligence, which may enable more objective intraoperative decision making based on accumulated data [51]. In this study, fluorescence signals were evaluated by more than two board-certified HBP surgeons for sufficiency; however, this remains a qualitative assessment. Quantitative measurement of fluorescence intensity may help prevent postoperative thrombosis in patients with insufficient fluorescence signals, underscoring the need for standardized quantitative evaluation criteria for ICG fluorescence imaging. Third, blood flow assessment in reconstructed vessels and surrounding organs using ultrasound sonography was not sufficiently evaluated in this study. This is due to the fact that this is a retrospective study, which includes cases lacking sufficient objective data on results of intraoperative ultrasound sonography. Future studies should compare the findings of ultrasound sonography, which has been the primary tool for blood flow evaluation, with those of ICG fluorescence imaging. Furthermore, based on the results, a more accurate blood flow evaluation method that combines these two methods should be investigated.

## 5. Conclusions

ICG fluorescence imaging is valuable for decision-making and evaluating blood flow in HBP surgeries that involve the resection of major vessels, such as hepatic veins, hepatic arteries, and PVs, with consideration for their reconstruction.

## Figures and Tables

**Table 1:** Clinical characteristics, ICG fluorescence imaging findings, and postoperative outcomes.

Patient No.	Surgical Technique	Resected Vessel	Reconstructed Method	ICG Dosage (mg)	Fluorescence Signal in Reconstructed Vessel	Postoperative Stenosis/Occlusion
1	Hepatectomy	RHV	Type 4 (umbilical vein)	2.5 mg	+	−
2	Hepatectomy	MHV	Reconstruction avoided *	1.25 mg	ND	ND
3	PD	CHA SMV	Type 3 Type 3	2 mg	+ +	− −
4	Extrahepatic bile duct resection	RHA	Type 3	2.5 mg	− **	+
5	Hepatectomy	RHV	Type 4 (umbilical vein)	2.5 mg	−	+
6	Hepatectomy	AFV	Reconstruction avoided *	2.5 mg	ND	ND
7	TP	SMV	Type 3	2.5 mg	+	−
8	PD	SMV CA	Type 3 Type 3	2.5 mg	+ +	− −
9	DP	SMV	Type 3	2.5 mg	+	−
10	PD	SMV	Type 3	2.5 mg	+	−
11	PD	SMV	Type 3	2.5 mg	+	−
12	PD	SMV SpV	Type 3 Type 3	2.5 mg	+ −	− +
13	PD	RHA	Type 3	5 mg	+	−
14	PD	SMV	Type 3	2.5 mg	+	−
15	PD	SMV SpV	Type 3 Type 3	2.5 mg	+ +	+ +
16	PD	SMV PHA	Type 3 Type 3	2.5 mg	+ +	− −

ICG, indocyanine green; PD, pancreaticoduodenectomy; TP, total pancreatectomy; DP, distal pancreatectomy; RHV, right hepatic vein; MHV, middle hepatic vein; CHA, common hepatic artery; SMV, superior mesenteric vein; RHA, right hepatic artery; AFV, anterior fissure vein; CA, celiac artery; SpV, splenic vein; PHA, proper hepatic artery; ND, not determined. * Vascular reconstruction was avoided because the targeted hepatic blood flow remained preserved without the need for reconstruction. ** A re-do anastomosis after reconstruction was performed. The sufficient fluorescence signal observed in the reconstructed vessel following the re-do anastomosis.

## Data Availability

All data generated or analyzed in this study are included in the article. Further inquiries can be directed to the corresponding author.

## Supplementary Materials

The following supporting information can be downloaded at https://doi.org/10.5281/zenodo.14942607 (accessed on 25 February 2025)—Video S1: Summarized operative movie in a patient who underwent an extended left hemi-hepatectomy with MHV resection. ICG fluorescence imaging showed only mild hepatic congestion and avoided reconstruction. Video S2: Summarized operative movie in a patient who underwent partial hepatectomy with RHV resection. ICG fluorescence imaging showed insufficient fluorescence at the site of vascular reconstruction. However, hepatic congestion improved over time, and no re-do anastomosis was performed.

## Abbreviations

The following abbreviations are used in this manuscript:

AFV	Anterior fissure vein
CA	Celiac artery
CHA	Common hepatic artery
CT	Computed tomography
DP	Distal pancreatectomy
ISGPS	International Study Group of Pancreatic Surgery
MHV	Middle hepatic vein
PHA	Proper hepatic artery
PV	Portal vein
